# Consumer Responses to Flexitarian Meals in Taiwan: Resistance-Based and Ethical–Evaluative Pathways

**DOI:** 10.3390/foods15203675

**Published:** 2026-10-16

**Authors:** Chun-Chieh Ma, Hsiao-Ping Chang

**Affiliations:** 1 Department of Public Administration and Management, National University of Tainan, No. 33, Sec. 2, Shu-Lin St., Tainan 70005, Taiwan; ccma@mail.nutn.edu.tw; 2 Department of Health Industry Technology Management, Chung Shan Medical University, No. 110, Sec. 1, Jianguo N. Rd., Taichung City 40201, Taiwan; 3 Department of Medical Management, Chung Shan Medical University Hospital, No. 110, Sec. 1, Jianguo N. Rd., Taichung City 40201, Taiwan

**Keywords:** flexitarian meals, meat reduction, food choice, image-related concerns, animal-welfare-related food-choice orientation, pro-environmental meal-choice behavior, perceived environmental severity, sustainable diets

## Abstract

Flexitarian meals were operationalized in this study as a heterogeneous set of meal-level options within a flexible meat-reduction context rather than as a standardized product category. Drawing on complementary perspectives informed by Innovation Resistance Theory (IRT) and the Value-Attitude-Behavior (VAB) framework, this cross-sectional study examined image-related concerns (IB), animal-welfare-related food-choice orientation (AWFO), support for flexitarian dietary transition (SFDT), purchase intention (PI), and self-reported pro-environmental meal-choice behavior (PEMCB). A convenience sample of 608 Taiwanese adults was surveyed from 4 to 10 May 2026. Structural equation modeling examined direct and indirect associations among the focal constructs, whereas separate hierarchical regression analyses using standardized observed composite scores tested the moderating roles of food-related pro-environmental orientation (FPEO) and perceived environmental severity (PS). IB was negatively associated with PI, whereas AWFO was positively associated with SFDT. SFDT was positively associated with both PI and PEMCB, while PI was not independently associated with PEMCB in the structural model. Bootstrap analyses further indicated significant indirect associations of AWFO with PI and PEMCB through SFDT. In the separate regression analyses, FPEO did not significantly moderate the hypothesized relationships, whereas the PS interactions were statistically significant but modest in magnitude. Small to modest age-group differences were observed among respondents aged 18–31, 32–45, and 46–61 years. Given the cross-sectional design, adapted self-report measures, heterogeneous meal examples, and non-probability sampling, the findings should be interpreted as associations rather than causal effects.

## 1. Introduction

The transition toward more sustainable food systems has increased interest in dietary patterns that reduce environmental pressures while remaining acceptable and feasible for consumers. Reducing meat consumption is one pathway toward improving the environmental sustainability of diets [1,2,3,4]. Flexitarian diets are generally characterized by intentional and flexible reductions in meat intake rather than complete avoidance of animal-derived foods [5,6,7,8]. In the present study, however, “flexitarian meals” were operationalized for survey purposes as a heterogeneous set of meal-level options situated within a flexible meat-reduction context. The questionnaire examples were intended to illustrate this choice space rather than define a single standardized product category. Accordingly, the term “flexitarian meal” is used here as a study-specific operational label for meal-level choices within a broader meat-reduction context, rather than as a uniform food category or as a marker of respondents’ dietary identity.

Taiwan provides a relevant setting for examining flexible meat reduction because substantial availability of animal-source foods coexists with established vegetarian dietary practices. Official statistics indicate that Taiwan’s annual per-capita meat food supply was 86.8 kg in 2024 [9]. Vegetarian dietary practices have also been documented among Taiwanese Buddhist volunteers [10]. This combination provides a useful context for examining consumer responses to flexible meat reduction without implying that flexitarianism has a known population prevalence in Taiwan.

Consumer responses to flexitarian meals may involve both resistance and supportive evaluations. Innovation Resistance Theory (IRT) provides a framework for understanding resistance to unfamiliar products or practices [11,12], whereas the Value-Attitude-Behavior (VAB) framework offers a complementary perspective on how value-related orientations may be associated with evaluations and consumer responses [13]. In this study, the two perspectives are treated as complementary rather than as a fully integrated theoretical system. The IRT-informed pathway focuses on concerns related to processing, definitional ambiguity, and labeling. The VAB-informed pathway is interpreted more cautiously as an ethical/evaluative pathway, linking animal-welfare-related food-choice orientation (AWFO) with support for flexitarian dietary transition (SFDT). AWFO is not treated as a pure measure of abstract personal values because its retained items incorporate purchasing, choosing, and preference statements.

Accordingly, this study examines the associations among image-related concerns (IB), AWFO, SFDT, purchase intention (PI), and self-reported pro-environmental meal-choice behavior (PEMCB). Food-related pro-environmental orientation (FPEO) and perceived environmental severity (PS) are examined as contextual moderators in separate observed-composite regression analyses, while age-group differences are explored as a supplementary source of consumer heterogeneity. This framework allows resistance-related and ethical/evaluative associations to be examined within the same food-choice context while maintaining clear distinctions among the constructs, their behavioral referents, and the analytical approaches used to test them.

## 2. Literature Review and Hypothesis Development

### 2.1. Flexitarian Meal Choice

Flexitarian diets have emerged as a pragmatic approach to reducing meat consumption without requiring complete adherence to vegetarian or vegan dietary patterns. Rather than eliminating meat entirely, flexitarianism emphasizes gradual and flexible reductions in meat intake, allowing consumers to modify established eating practices incrementally [5,6,7,8]. A flexitarian meal, as used in this study, is therefore a study-specific meal-level choice considered within this broader reduced-meat dietary context rather than a standardized food category, a separate dietary pattern, or a label for respondents’ dietary identity.

Consumer responses to flexitarian and other reduced-meat foods are heterogeneous. Previous research indicates that ethical considerations, environmental concerns, health motivations, sensory preferences, familiarity, habits, and contextual constraints can all be associated with willingness to adopt plant-rich or lower-meat dietary alternatives [14,15,16,17,18,19,20,21,22,23,24]. Thus, flexitarian meal choice cannot be explained solely by favorable motivations; consumers may simultaneously perceive benefits while experiencing uncertainty or resistance toward unfamiliar dietary options.

To capture these complementary processes, the present study draws on two theoretical perspectives. IRT provides a basis for examining resistance that may inhibit acceptance of unfamiliar food options [11,12], whereas the VAB framework provides a conceptual basis for considering how value-related orientations may be associated with supportive evaluations and consumer responses [13]. The model therefore examines an IRT-informed resistance pathway alongside a VAB-informed ethical/evaluative pathway, without proposing a fully integrated IRT–VAB theory.

### 2.2. IRT-Informed Resistance Pathway: Image-Related Concerns and Purchase Intention

Innovation Resistance Theory explains why consumers may resist the adoption of new products or consumption practices even when such innovations offer potential benefits [11,12]. Resistance can arise when an unfamiliar option conflicts with existing habits, expectations, beliefs, or category perceptions. In the present study, the retained indicators capture concerns about processing, ambiguity in flexitarian guidelines, and ingredient labeling. Because this operationalization extends beyond a narrow classical image barrier, the construct is referred to more cautiously as image-related concerns (IB).

Previous food-related studies indicate that image-related resistance is negatively associated with purchase intention toward sustainable and innovative foods [25,26]. Similarly, plant-based alternatives perceived as unnatural, highly processed, or difficult to categorize tend to receive lower consumer acceptance and purchase intention [27,28]. Flexitarian meals may generate comparable uncertainty when consumers are unclear about their ingredients, processing, labeling, or dietary identity. Accordingly, greater image-related concerns are expected to be associated with lower purchase intention.

**H1.**  *Image-related concerns are negatively associated with consumers’ purchase intention toward flexitarian meals.*

### 2.3. VAB-Informed Ethical–Evaluative Pathway: Animal-Welfare-Related Food-Choice Orientation and Support for Flexitarian Dietary Transition

The Value-Attitude-Behavior (VAB) framework proposes that relatively enduring personal values may be associated with evaluations of specific objects or behaviors, which in turn may relate to intentions and behavioral responses [13]. Prior applications of the VAB framework in plant-based and vegan food contexts have linked personal and ethical values with dietary evaluations and behavioral responses [29,30,31]. Conventional attitude measures, however, typically assess an individual’s evaluative response toward a specific object or behavior [32,33]. In contrast, the two retained evaluative items in the present study assess support for promoting flexitarian diets and addressing problems associated with meat consumption in Taiwan. This construct is therefore interpreted as support for flexitarian dietary transition (SFDT) rather than as a conventional personal attitude toward consuming flexitarian meals.

Animal-welfare-related food-choice orientation (AWFO) reflects the extent to which concern for animal welfare is expressed through food-related purchasing, choosing, and preference tendencies. The retained indicators refer to purchasing brands labeled as not involving animal slaughter, choosing brands and meals that do not involve animal testing, and preferring ethically produced meals. Accordingly, AWFO should not be interpreted as a pure measure of abstract animal welfare values. Rather, it represents a narrower animal-welfare-related orientation expressed within food-choice contexts. Previous research nevertheless indicates that animal-related ethical concerns and orientations are associated with meat reduction, vegetarian choices, and welfare-oriented food purchasing [34,35,36,37,38,39].

Flexitarian meals can provide a way to reduce reliance on meat without requiring complete meat avoidance. Consumers with a stronger animal-welfare-related food-choice orientation may therefore report greater support for a flexitarian dietary transition.

**H2.**  *Animal-welfare-related food-choice orientation is positively associated with support for flexitarian dietary transition.*

### 2.4. From Support to Choice: Purchase Intention and Pro-Environmental Meal-Choice Behavior

Purchase intention reflects prospective willingness or readiness to choose or purchase a product in the future [40,41,42]. In the present study, PI refers specifically to willingness to choose flexitarian meals. PEMCB was assessed using two self-reported items that share an environmentally oriented meal-choice focus but differ partially in their behavioral referents. PB1 refers specifically to choosing a lower-impact option when purchasing flexitarian meals, whereas PB2 refers more generally to choosing a lower-impact option among similar meals. Accordingly, PEMCB is interpreted as a brief, behavior-proximal self-report measure with partially different referents rather than as a broad or fully homogeneous pro-environmental behavioral construct.

PI and PEMCB are related but not fully aligned in behavioral referent. PI captures prospective willingness to choose flexitarian meals, whereas PEMCB captures self-reported environmentally oriented meal choices across two partially different choice contexts. H3 was therefore retained to examine whether these related food-choice constructs are positively associated. Because the measures are not behaviorally identical, any observed association should be interpreted with attention to their partial referent mismatch rather than as a direct test of a conventional intention–behavior sequence.

**H3.**  *Purchase intention toward flexitarian meals is positively associated with self-reported pro-environmental meal-choice behavior.*

SFDT reflects support for promoting flexitarian diets and addressing problems associated with meat consumption in Taiwan. Although this differs from a conventional personal attitude scale, stronger support for dietary transition may still be associated with prospective willingness to choose flexitarian meals and with self-reported environmentally oriented meal-choice behavior. Previous plant-based food studies have likewise linked favorable evaluative orientations with behavioral intentions and related consumer responses [30,31,43].

**H4.**  *Support for flexitarian dietary transition is positively associated with self-reported pro-environmental meal-choice behavior.*

**H5.**  *Support for flexitarian dietary transition is positively associated with purchase intention toward flexitarian meals.*

### 2.5. Environmental Context as a Moderator: Food-Related Pro-Environmental Orientation and Perceived Environmental Severity

General environmental concern is typically conceptualized as a broad orientation toward environmental issues [44]. In contrast, the retained items in this study refer specifically to food-related pro-environmental tendencies, including reducing purchases of environmentally harmful meat-based meals, seeking environmentally friendly meal options, and encouraging similar choices among others. Accordingly, the construct is interpreted as food-related pro-environmental orientation (FPEO) rather than general environmental concern.

FPEO is relatively close to food-choice behavior and may therefore be related directly to food-choice outcomes as well as potentially moderating other associations. Because of this behavioral proximity, its conceptual distinction from PI and PEMCB is evaluated and interpreted cautiously.

In the separate observed-composite moderation analyses, FPEO was examined as a moderator of the IB–PI and AWFO–SFDT associations. From an IRT-informed perspective, FPEO may alter the association between image-related concerns and PI; from a VAB-informed ethical/evaluative perspective, it may alter the association between AWFO and SFDT.

**H6.**  *Food-related pro-environmental orientation moderates the negative association between image-related concerns and purchase intention toward flexitarian meals.*

**H7.**  *Food-related pro-environmental orientation moderates the positive association between animal-welfare-related food-choice orientation and support for flexitarian dietary transition.*

Perceived environmental severity (PS) is conceptually distinct from FPEO. It refers to respondents’ appraisal of the seriousness, harmfulness, and increasing consequences of environmental degradation [45,46,47]. Within threat-appraisal perspectives, perceived severity reflects the extent to which environmental problems are viewed as sufficiently serious to warrant attention. Previous sustainable-food research suggests that PS does not necessarily operate as a strong direct predictor of dietary intention; its role may instead depend on the broader decision context.

When environmental degradation is perceived as more serious, consumers may scrutinize sustainability-related food choices more closely. Under such conditions, ambiguity surrounding an environmentally positioned food option may become more consequential, while ethical considerations may also become more salient.

For flexitarian meals, the negative association between IB and PI is therefore expected to be stronger at higher levels of PS, whereas the positive association between AWFO and SFDT is expected to become stronger as PS increases.

**H8.**  *Perceived environmental severity moderates the negative association between image-related concerns and purchase intention toward flexitarian meals.*

**H9.**  *Perceived environmental severity moderates the positive association between animal-welfare-related food-choice orientation and support for flexitarian dietary transition.*

**Figure 1:**
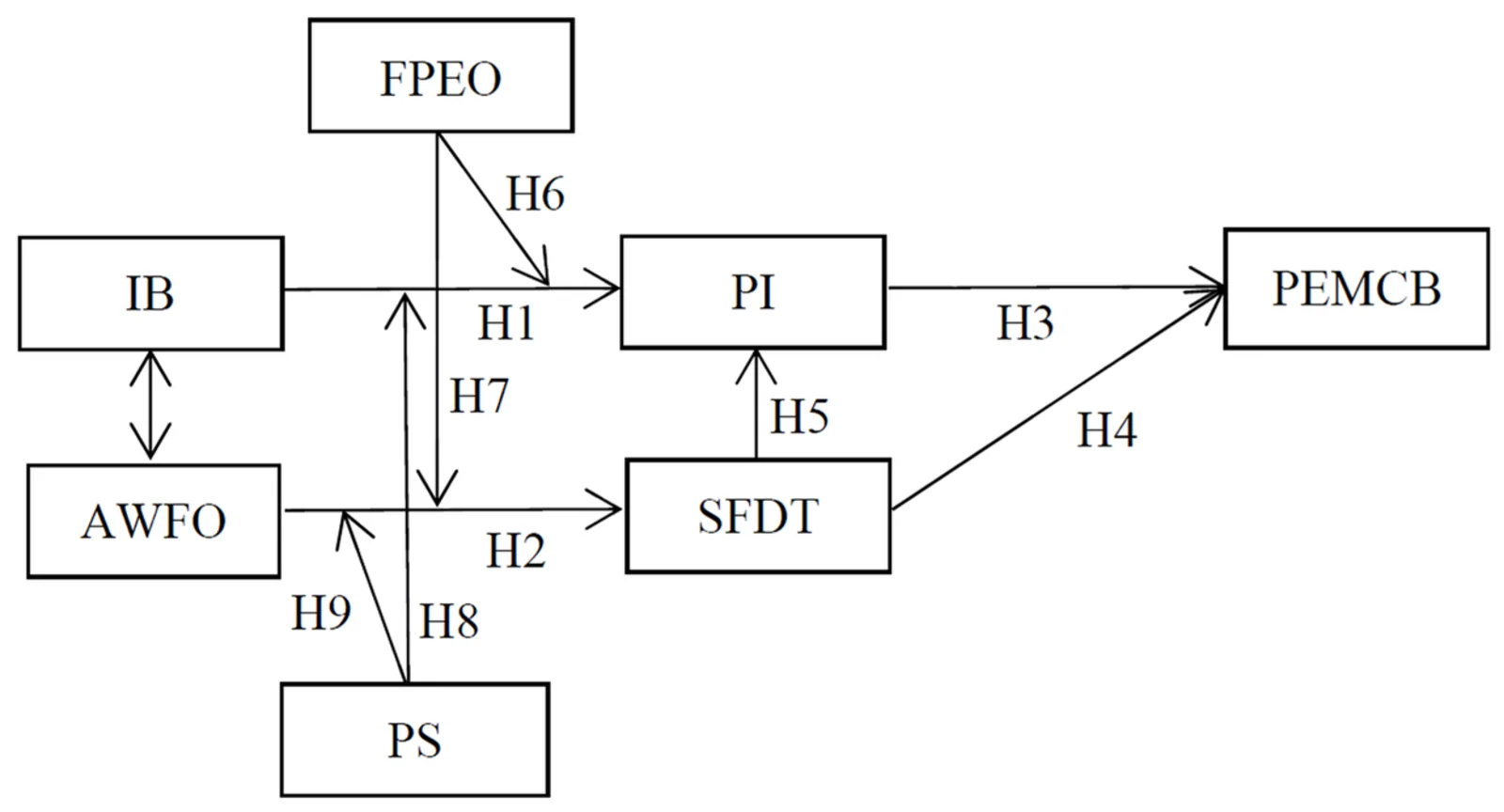
Research framework showing complementary IRT-informed resistance and VAB-informed ethical/evaluative pathways. The double-headed arrow between IB and AWFO denotes covariance. The moderating roles of FPEO and PS were tested separately using observed-composite regression analyses and were not estimated as latent SEM interactions. IB = Image-Related Concerns; AWFO = Animal-Welfare-Related Food-Choice Orientation; SFDT = Support for Flexitarian Dietary Transition; PI = Purchase Intention; PEMCB = Pro-Environmental Meal-Choice Behavior; FPEO = Food-Related Pro-Environmental Orientation; PS = Perceived Environmental Severity.

The latent structural model also implies indirect associations linking AWFO with PI and PEMCB through SFDT. These indirect associations were evaluated using bootstrap confidence intervals as supplementary tests of the structural model. Given the cross-sectional design, they are interpreted as statistical indirect associations rather than evidence of causal mediation. Figure 1 summarizes the direct structural associations and the separately tested moderating relationships examined in the study.

## 3. Materials and Methods

### 3.1. Sample, Pilot Testing, and Data Collection

Before the formal survey, a pilot test was conducted on 2–3 May 2026 using convenience sampling and both online and paper questionnaires. Of the 76 questionnaires returned, eight were excluded because of substantial missing responses or response patterns indicative of careless responding, including long strings of identical responses and identical response selections on positively worded and reverse-worded items within the same construct, leaving 68 usable questionnaires (89.5%). The pilot instrument initially contained 42 measurement items across seven constructs: five IB items, six PI items, six PEMCB items, nine animal-welfare-related items, two SFDT items, nine FPEO items, and five PS items. Item screening considered factor loadings and item-content clarity, and items with factor loadings below 0.50 were not retained. Using the original pilot-questionnaire numbering, the items removed were IB3 and IB5; PI3; PB3–PB6; AWV1–AWV3 and AWV7–AWV9; EC1–EC4, EC8, and EC9; and PS3. Both SFDT items were retained. The retained items were subsequently renumbered within their final constructs for presentation in the manuscript. Principal-axis factoring was used for the pilot item analysis. The archived pilot documentation did not record the rotation method. Following item screening and refinement, Cronbach’s α increased from 0.578 to 0.729 for IB, from 0.761 to 0.925 for PI, from 0.714 to 0.882 for PEMCB, from 0.871 to 0.878 for the construct now labeled AWFO, from 0.772 to 0.857 for FPEO, and from 0.680 to 0.928 for PS; the reliability coefficient for SFDT remained unchanged at 0.766. The retained 22-item set showed an overall KMO value of 0.712, and Bartlett’s test of sphericity was significant, χ^2^(231) = 801.852, *p* < 0.001. Principal-axis factoring yielded loadings ranging from 0.537 to 0.880 for the retained items. The final 22-item questionnaire was established after pilot testing and item refinement and before formal data collection; none of the pilot respondents were included in the formal analytic sample.

The formal survey used a cross-sectional, non-probability convenience-sampling design. The questionnaire was distributed primarily through LINE and Facebook, with additional dissemination through student networks and personal contacts. Formal data collection was conducted from 4 to 10 May 2026. A total of 701 questionnaires were returned. After screening, 93 responses were excluded because of substantial missing responses or response patterns indicative of careless responding, including long strings of identical responses and identical response selections on positively worded and reverse-worded items within the same construct. The final analytic sample therefore comprised 608 usable questionnaires, yielding a usable-questionnaire rate of 86.7%. The archived screening record preserved the aggregate number of excluded responses but did not retain a reliable category-specific breakdown of the 93 exclusions.

All participants were aged 18 years or older, were informed of the study purpose, and provided informed consent. The study was approved by the Institutional Review Board of Chung Shan Medical University, Taiwan (IRB No. CS2-26194; approval date: 2 May 2026). Formal data collection therefore occurred after IRB approval.

For age-group comparisons, respondents were classified using the original grouped-age variable recorded in the dataset. The original age categories were 18–21, 22–26, 27–31, 32–38, 39–45, 46–53, and 54–61 years. These categories were combined into three broader age groups for comparative analysis: 18–31 years (*n* = 293), 32–45 years (*n* = 150), and 46–61 years (*n* = 165). Because exact birth year was not collected, these comparisons are interpreted strictly as age-group differences rather than as generational or cohort effects.

Women accounted for 65.0% of the sample (*n* = 395) and men for 35.0% (*n* = 213). Most respondents had completed college or university education (72.5%), while 15.5% held a master’s degree or above. Regarding dietary preference, 45.2% reported consuming non-vegetarian meals, 4.6% vegetarian meals, and 50.2% both vegetarian and non-vegetarian meals. The latter category was not treated as equivalent to flexitarian status because intentional meat reduction, meat-consumption frequency, and flexitarian self-identification were not assessed. Income was not collected in the original questionnaire and therefore could not be reported retrospectively. Sample characteristics are presented in Table 1.

### 3.2. Questionnaire and Measurement

The questionnaire administered to participants was in Chinese. An English version was prepared for manuscript reporting and peer review; however, no formally documented back-translation procedure was available. Before responding to the measurement items, participants were provided with a study-specific explanation that positioned flexitarian meals between conventional meat-based and fully vegan meal choices. Examples included light meals, vegetable-based meals, plant-based meat, cultivated meat, and non-vegan vegetarian options such as lacto-ovo vegetarian meals. This description reflects the operational definition used in the questionnaire and should not be interpreted as a universal taxonomy of flexitarian foods.

All retained items were measured on seven-point Likert scales ranging from 1 (strongly disagree) to 7 (strongly agree). The final measurement model comprised seven constructs and 22 items: image-related concerns (IB), purchase intention (PI), pro-environmental meal-choice behavior (PEMCB), animal-welfare-related food-choice orientation (AWFO), support for flexitarian dietary transition (SFDT), food-related pro-environmental orientation (FPEO), and perceived environmental severity (PS).

For traceability, the original dataset item codes PB, AWV, AT, and EC are retained in the tables and Supplementary Materials, although the corresponding construct labels are reported as PEMCB, AWFO, SFDT, and FPEO. IB was measured using three context-specific items informed by Laukkanen and Kiviniemi; PI using five items adapted from prior behavioral-intention measures; PEMCB using two items adapted from Rossi and Rivetti; AWFO using three items informed by Michaelidou and Hassan; SFDT using two context-adapted items informed by Taufique et al.; FPEO using three food-choice items adapted from Yadav and Pathak; and PS using four items informed by Sharma et al., and recontextualized to environmental degradation.

For interpretive clarity, construct labels were aligned with the content of the retained items. This relabeling did not alter the questionnaire items, participant responses, scoring procedures, or underlying data. Complete item wording is provided in Table 2, and item-level descriptive statistics are reported in Supplementary Table S4.

### 3.3. Statistical Analysis

Statistical analyses were conducted using IBM SPSS Statistics version 29 and AMOS version 27. Descriptive statistics were used to summarize sample characteristics and study variables. For descriptive analyses, Pearson correlations, moderation analyses, and age-group comparisons, observed composite scores were calculated as the arithmetic mean of the retained items within each construct.

Confirmatory factor analysis (CFA) and structural equation modeling (SEM) were estimated using maximum likelihood (ML). Multivariate normality and potentially influential observations were assessed using AMOS multivariate-kurtosis and Mahalanobis-distance diagnostics. Because substantial multivariate non-normality was detected, the ML chi-square statistic and conventional standard errors were interpreted cautiously. Model evaluation therefore emphasized multiple fit indices rather than the chi-square statistic alone, and bias-corrected bootstrap confidence intervals based on 5000 resamples were used for the reported direct and indirect structural associations. No robust ML estimator was applied; accordingly, residual sensitivity to non-normality is acknowledged as a limitation. The final analytic dataset contained complete data for all variables included in the reported analyses; therefore, no missing-data imputation was performed.

Measurement-model fit was evaluated primarily using χ^2^/df, the comparative fit index (CFI), Tucker–Lewis index (TLI), standardized root mean square residual (SRMR), and root mean square error of approximation (RMSEA), with the goodness-of-fit index (GFI), adjusted goodness-of-fit index (AGFI), and normed fit index (NFI) additionally reported for comparability with the original analytical framework [56,57,58]. Internal consistency and convergent validity were assessed using Cronbach’s α, standardized factor loadings, composite reliability (CR), and average variance extracted (AVE) [59,60]. Discriminant validity was evaluated using the Fornell–Larcker criterion [60] and heterotrait–monotrait ratios (HTMT) [61] (Supplementary Table S1). Common-method variance was assessed using Harman’s single-factor test and a single-factor CFA. These analyses were treated as diagnostic checks and not as evidence that common-method variance was absent.

SEM was used to estimate the direct structural associations among IB, AWFO, SFDT, PI, and PEMCB. Both unstandardized coefficients (B) and standardized coefficients (β) are reported. Bias-corrected 95% bootstrap confidence intervals based on 5000 resamples were obtained for standardized direct-path estimates and indirect associations. Given the cross-sectional design, indirect findings are interpreted as statistical indirect associations rather than causal mediation effects.

Hierarchical ordinary least-squares regression analyses were used to test the moderating roles of FPEO and PS separately from the latent SEM, using observed composite scores. For each moderation model, the outcome, focal predictor, and moderator composite scores were standardized as z scores before estimation, and interaction terms were constructed as products of the corresponding standardized predictor and moderator. Incremental explained variance (ΔR^2^), interaction coefficients, standard errors, 95% confidence intervals, and exact *p*-values are reported where applicable. Significant PS interactions were examined using simple slopes at one standard deviation below the mean, at the mean, and one standard deviation above the mean. Because these analyses use observed composite scores rather than latent variables, their coefficient magnitudes should not be directly compared with those obtained from the latent SEM.

Before age-group comparisons, multigroup CFA was conducted to test configural, metric, and scalar measurement invariance across the three age groups defined in Section 3.1 (Supplementary Table S2). Changes in CFI and RMSEA were used as practical criteria for evaluating measurement invariance [62]. One-way ANOVA was subsequently used to compare observed composite scores across age groups. Eta-squared (η^2^) was reported as an effect-size measure, and Scheffé post hoc tests were conducted following significant omnibus tests. Statistical significance was set at *p* < 0.05.

## 4. Results

### 4.1. Measurement Model Assessment

The seven-construct, 22-item measurement model demonstrated acceptable fit: χ^2^(188) = 737.766, *p* < 0.001, χ^2^/df = 3.924, GFI = 0.894, AGFI = 0.858, NFI = 0.923, TLI = 0.927, CFI = 0.941, SRMR = 0.038, and RMSEA = 0.069 (90% CI [0.064, 0.075]). Although GFI and AGFI were slightly below 0.90, the overall pattern of CFI, TLI, SRMR, and RMSEA values indicated acceptable measurement-model fit. The CFA specified seven correlated factors, with each item loading only on its intended construct; no correlated residuals were introduced solely to improve model fit. Detailed CFA measurement-model estimates and latent construct correlations are provided in Supplementary Table S3.

Cronbach’s α values ranged from 0.729 to 0.928, CR values from 0.732 to 0.926, and standardized factor loadings from 0.603 to 0.937. AVE exceeded 0.50 for six constructs. IB had an AVE of 0.479, indicating marginal rather than strong convergent validity. Its α (0.729), CR (0.732), and significant factor loadings (0.603–0.749) supported retention of the three-item specification, although the construct was interpreted cautiously. Removing the weakest indicator did not provide a compelling improvement in reliability and would have reduced the construct to two indicators. The factor loadings, reliability estimates, and convergent validity results are presented in Table 3.

Discriminant validity was examined at both the latent and observed-score levels. The largest latent correlation was between PI and SFDT (r = 0.751), which remained below the square roots of AVE for PI (0.843) and SFDT (0.792). The corresponding Pearson correlation between observed composite scores was r = 0.626. All HTMT values were below 0.85, with the largest value observed for PI–SFDT (0.745; Supplementary Table S1). The means, standard deviations, and Pearson correlation coefficients based on observed composite scores are presented in Table 4.

Harman’s unrotated analysis yielded five factors with eigenvalues greater than 1, with the first factor accounting for 39.64% of the total variance. A single-factor CFA showed poor fit, χ^2^(209) = 4517.203, *p* < 0.001, CFI = 0.537, TLI = 0.488, SRMR = 0.138, and RMSEA = 0.184, which was inconsistent with a single-factor representation of the data. These diagnostics do not rule out common-method variance. AMOS also indicated substantial multivariate non-normality (multivariate kurtosis = 134.583, critical ratio = 73.473). Mahalanobis-distance diagnostics were examined for potentially influential cases, and inferential findings were interpreted cautiously. Consistent with the analytical approach described in Section 3.3, model fit was therefore interpreted using multiple fit indices rather than the chi-square statistic alone, and bias-corrected bootstrap confidence intervals were used for the reported structural estimates; residual sensitivity to non-normality remains a limitation. 

### 4.2. Structural Equation Modeling and Hypothesis Testing

**Figure 2:**
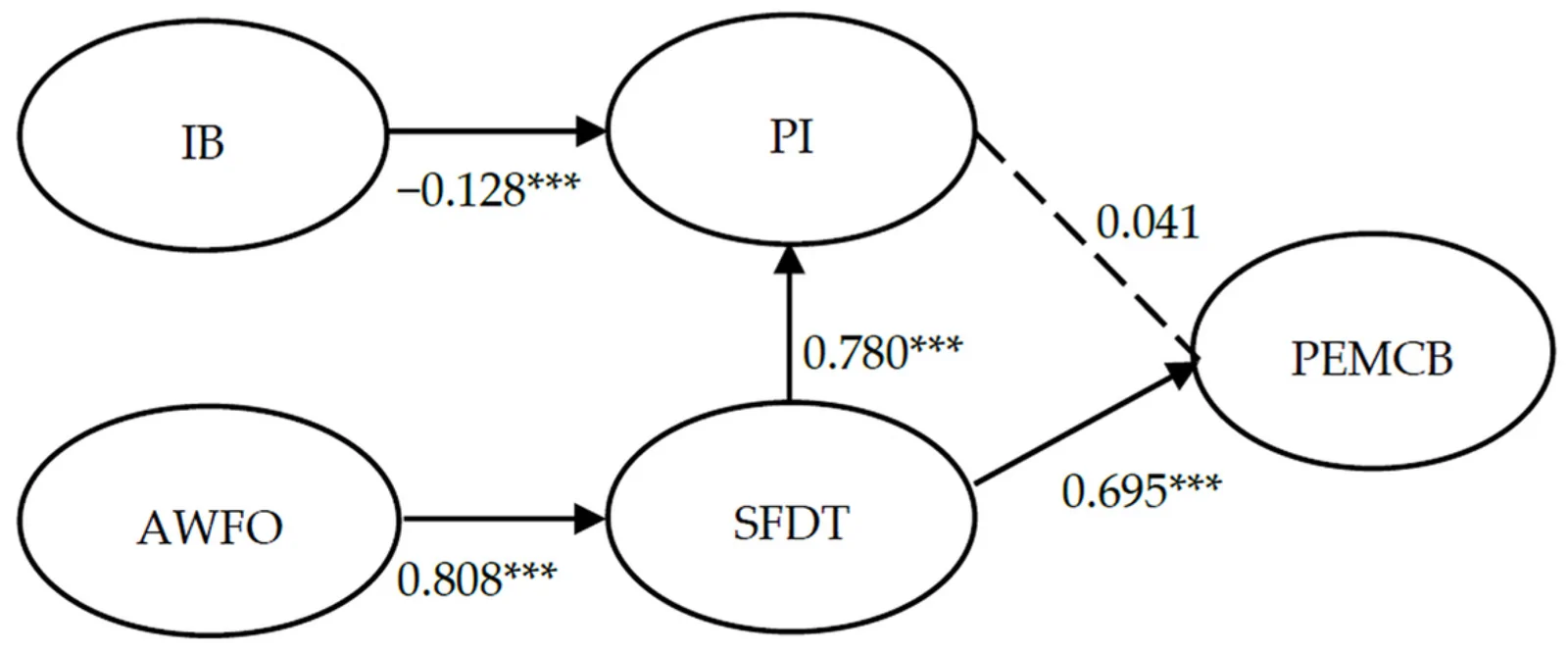
Standardized path coefficients of the structural model. Solid lines indicate statistically significant associations, whereas the dashed PI → PEMCB path indicates a non-significant association; *** *p* < 0.001.

The structural model demonstrated acceptable fit: χ^2^(84) = 244.457, *p* < 0.001, χ^2^/df = 2.910, GFI = 0.947, AGFI = 0.925, NFI = 0.957, RFI = 0.946, IFI = 0.971, TLI = 0.964, CFI = 0.971, SRMR = 0.033, and RMSEA = 0.056 (90% CI [0.048, 0.064]). Figure 2 presents the standardized path coefficients, whereas Table 5 reports both unstandardized and standardized estimates together with bootstrap confidence intervals.

IB was negatively associated with PI (B = −0.152, SE = 0.043, β = −0.128, *p* < 0.001), supporting H1. AWFO was positively associated with SFDT (B = 0.676, SE = 0.043, β = 0.808, *p* < 0.001), supporting H2. PI was not significantly associated with PEMCB (B = 0.041, SE = 0.076, β = 0.041, *p* = 0.588); thus, H3 was not supported. SFDT was positively associated with PEMCB (B = 0.833, SE = 0.107, β = 0.695, *p* < 0.001) and PI (B = 0.925, SE = 0.061, β = 0.780, *p* < 0.001), supporting H4 and H5, respectively.

Bootstrap analyses based on 5000 resamples showed significant positive indirect associations from AWFO to PI through SFDT (standardized indirect effect = 0.630, bias-corrected 95% CI [0.563, 0.695]) and from AWFO to PEMCB through SFDT (0.562, 95% CI [0.400, 0.719]). The serial AWFO → SFDT → PI → PEMCB indirect association was not significant (0.026, 95% CI [−0.092, 0.138]), nor was the SFDT → PI → PEMCB indirect association (0.032, 95% CI [−0.112, 0.172]). The total indirect association from AWFO to PEMCB was significant (0.588, 95% CI [0.499, 0.669]). These findings are interpreted as statistical indirect associations rather than evidence of causal mediation.

### 4.3. Moderation Analysis

Separate hierarchical ordinary least-squares regression analyses examined the moderating roles of FPEO and PS using standardized observed composite scores; these interaction models were estimated separately from the latent SEM.

FPEO did not significantly moderate either hypothesized relationship. For the IB × FPEO interaction predicting PI, B = −0.031, SE = 0.029, β = −0.037, 95% CI [−0.089, 0.027], ΔR^2^ = 0.001, ΔF = 1.109, *p* = 0.293; thus, H6 was not supported (Table 6). Similarly, the AWFO × FPEO interaction predicting SFDT was not significant, B = 0.036, SE = 0.027, β = 0.044, 95% CI [−0.017, 0.089], ΔR^2^ = 0.002, ΔF = 1.821, *p* = 0.178; H7 was not supported (Table 7).

Within the hierarchical regression predicting PI, adding FPEO was associated with an increase in explained variance of ΔR^2^ = 0.243. This finding is reported descriptively and does not imply that FPEO was tested as a direct predictor in the latent structural model.

**Figure 3:**
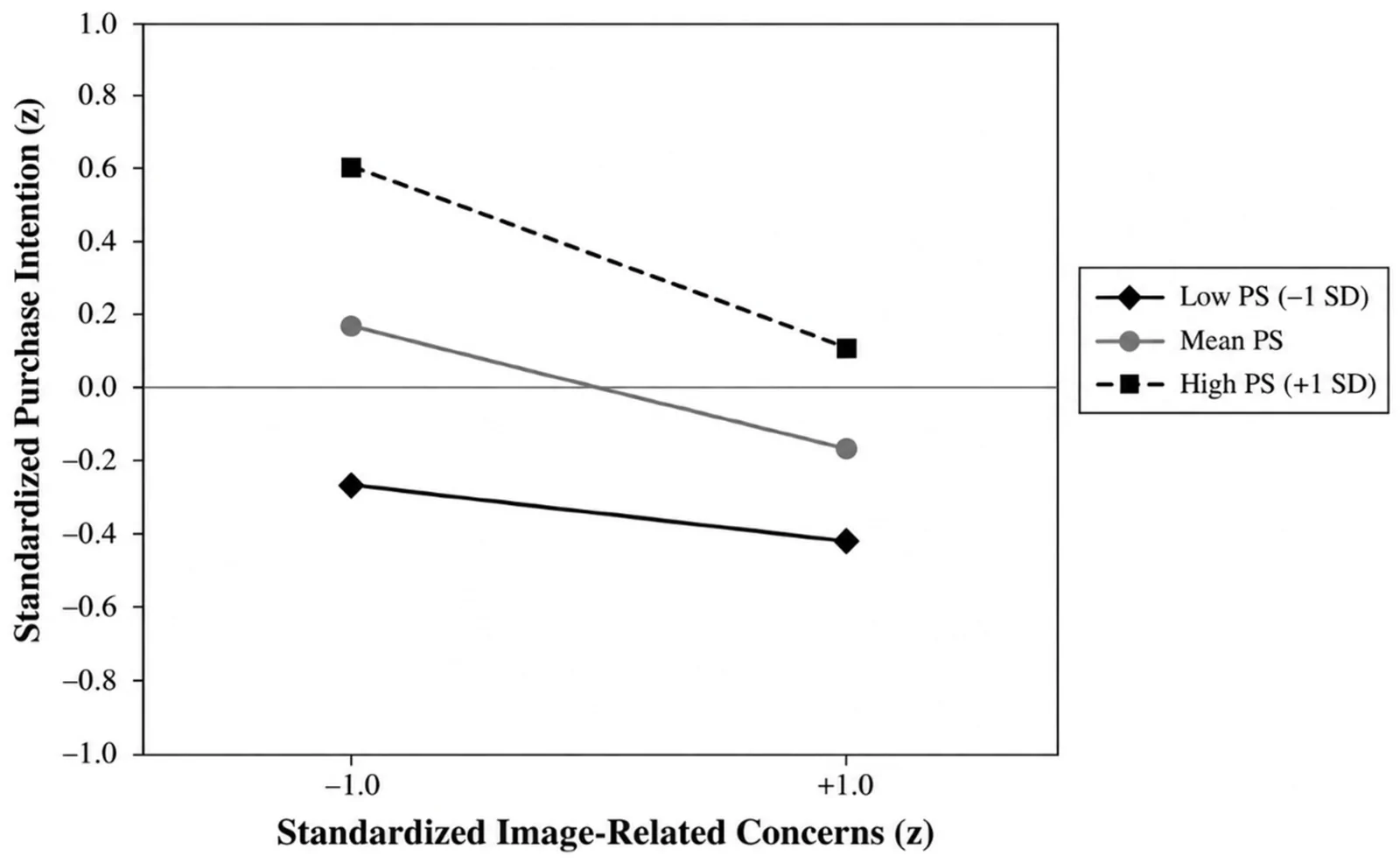
Moderating role of perceived environmental severity in the association between image-related concerns and purchase intention. Predicted standardized purchase intention is shown at standardized IB values of −1 and +1, with PS represented at −1 SD, the mean, and +1 SD. The simple slope was non-significant at low PS (−1 SD; *p* = 0.065) but significant at mean and high PS (both *p* < 0.001).

PS showed statistically significant but modest moderation. For the IB × PS interaction predicting PI, the interaction was negative and significant (B = −0.070, SE = 0.031, β = −0.087, 95% CI [−0.131, −0.010], ΔR^2^ = 0.007, ΔF = 5.199, *p* = 0.023), supporting H8 (Table 8). Simple-slope analysis showed that the association between IB and PI was not statistically significant at low PS (−1 SD; B = −0.094, SE = 0.051, 95% CI [−0.193, 0.006], *p* = 0.065), but was significant at the mean level of PS (B = −0.164, SE = 0.038, 95% CI [−0.238, −0.090], *p* < 0.001) and at high PS (+1 SD; B = −0.234, SE. = 0.047, 95% CI [−0.326, −0.142], *p* < 0.001). The moderating effect of perceived environmental severity on the relationship between image-related concerns and purchase intention is illustrated in Figure 3.

**Figure 4:**
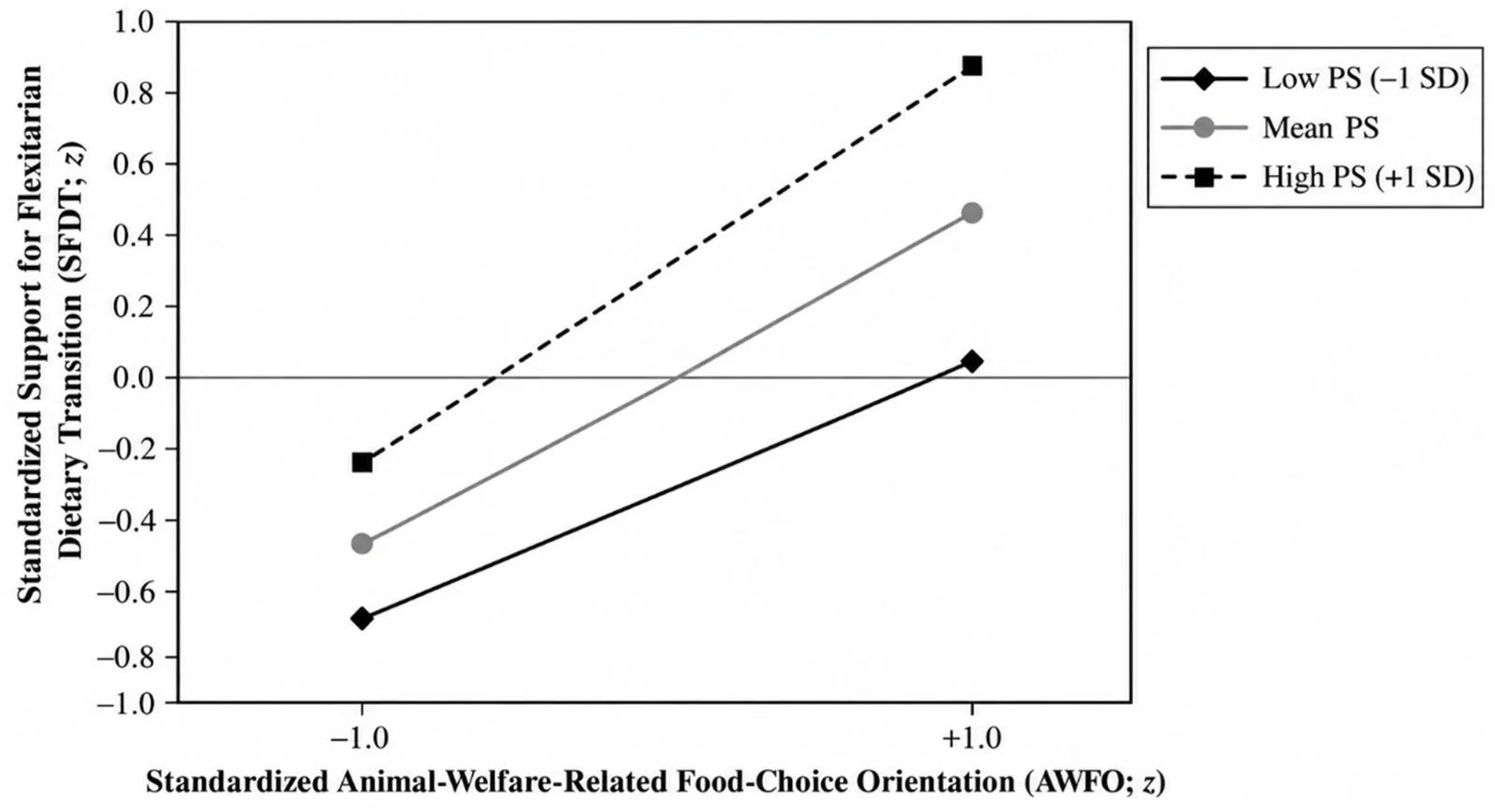
Moderating role of perceived environmental severity in the association between animal-welfare-related food-choice orientation and support for flexitarian dietary transition. Predicted standardized SFDT is shown at standardized AWFO values of −1 and +1, with PS represented at −1 SD, the mean, and +1 SD. All three simple slopes were positive and statistically significant (all *p* < 0.001).

For the AWFO × PS interaction predicting SFDT, the interaction was positive and significant (B = 0.076, SE = 0.027, β = 0.096, 95% CI [0.024, 0.129], ΔR^2^ = 0.008, ΔF = 8.099, *p* = 0.005), supporting H9 (Table 9). The moderating effect of perceived environmental severity on the association between animal-welfare-related food-choice orientation and support for flexitarian dietary transition is illustrated in Figure 4. 

Positive simple slopes were observed at low PS (−1 SD; B = 0.382, SE = 0.049, 95% CI [0.287, 0.478], *p* < 0.001), mean PS (B = 0.458, SE = 0.036, 95% CI [0.389, 0.528], *p* < 0.001), and high PS (+1 SD; B = 0.535, SE = 0.040, 95% CI [0.457, 0.613], *p* < 0.001). Because both interaction terms accounted for less than 1% additional explained variance, they are interpreted as modest contextual associations.

### 4.4. Age-Group Differences

Multigroup CFA supported configural, metric, and scalar measurement invariance across the three age groups (18–31 years, *n* = 293; 32–45 years, *n* = 150; and 46–61 years, *n* = 165; Supplementary Table S2). The configural model showed χ^2^ = 1341.544, df = 564, CFI = 0.921, TLI = 0.902, and RMSEA = 0.048. Constraining factor loadings to equality resulted in χ^2^ = 1407.271, df = 594, CFI = 0.917, TLI = 0.903, and RMSEA = 0.047, with ΔCFI = 0.004 and ΔRMSEA = 0.001. Adding equality constraints on item intercepts yielded χ^2^ = 1458.383, df = 624, CFI = 0.915, TLI = 0.905, and RMSEA = 0.047, with ΔCFI = 0.002 and ΔRMSEA < 0.001. Although the chi-square difference tests were significant for the metric model, Δχ^2^(30) = 65.727, *p* < 0.001, and the scalar model, Δχ^2^(30) = 51.112, *p* = 0.009, the small changes in CFI and RMSEA supported practical measurement invariance.

One-way ANOVA based on the observed composite scores identified significant age-group differences for IB, PI, PEMCB, AWFO, SFDT, and FPEO, whereas PS did not differ significantly across age groups (Table 10). Effect sizes were small to modest, with PEMCB showing the largest age-group effect (η^2^ = 0.061), followed by FPEO (η^2^ = 0.039) and IB (η^2^ = 0.037). Scheffé post hoc comparisons indicated that respondents aged 46–61 years reported higher PI, PEMCB, AWFO, SFDT, and FPEO scores than both younger age groups. For IB, respondents aged 32–45 and 46–61 years reported higher scores than those aged 18–31 years. Because exact birth year was not collected and the comparisons are cross-sectional, these findings are interpreted strictly as age-group differences rather than generational or cohort effects. Age, life stage, recruitment patterns, and other unmeasured characteristics may also have contributed to the observed differences.

## 5. Discussion

### 5.1. Resistance-Based and Ethical–Evaluative Pathways in Flexitarian Food Choice

The findings support two complementary pathways rather than a fully integrated IRT–VAB mechanism. In the IRT-informed pathway, image-related concerns showed a modest negative association with PI (β = −0.128). Because the retained IB items capture concerns related to processing, definitional ambiguity, and ingredient labeling, this finding is best interpreted as indicating that such concerns are associated with lower willingness to choose flexitarian meals rather than as a definitive test of a classical image barrier.

In the VAB-informed ethical/evaluative pathway, AWFO was strongly associated with SFDT (β = 0.808), and SFDT was positively associated with both PI (β = 0.780) and PEMCB (β = 0.695). Bootstrap analyses also showed significant indirect associations of AWFO with PI and PEMCB through SFDT. Because AWFO incorporates animal-welfare-related food-choice tendencies rather than abstract values alone, and SFDT reflects support for promoting flexitarian diets and addressing problems associated with meat consumption rather than a conventional personal attitude toward consuming a specific meal, these findings are best interpreted as an ethical/evaluative orientation-to-support pathway associated with intention and self-reported meal-choice tendencies, rather than as a strict temporal VAB sequence.

Although PI and PEMCB were positively correlated at the bivariate level (r = 0.526), the PI → PEMCB structural path was non-significant after SFDT was included in the model (β = 0.041, *p* = 0.588). The zero-order correlation indicates that PI and PEMCB covary without adjustment for the other modeled relationships, whereas the structural coefficient represents the unique association of PI with PEMCB conditional on the other paths in the model. The attenuation of this association is consistent with substantial shared variance involving SFDT, which was positively correlated with both PI (r = 0.626) and PEMCB (r = 0.524), in addition to the partially different behavioral referents of PI and PEMCB. Accordingly, the non-significant structural path should not be attributed primarily to referent mismatch. Rather, PI did not explain unique variation in PEMCB beyond the relationships captured by SFDT in the specified model. Given the cross-sectional design, this finding should not be interpreted as evidence for or against a temporal intention–behavior sequence.

Taken together, the findings indicate the coexistence of a modest resistance-related association and a stronger ethical/evaluative support pathway within the specified model. This complementary interpretation is more consistent with the estimated associations than a claim that IRT and VAB constitute a single integrated causal mechanism.

### 5.2. Environmental Context and Consumer Heterogeneity

The environmental analyses indicate distinct roles for FPEO and PS. FPEO did not significantly moderate either the IB–PI or AWFO–SFDT association. Within the hierarchical regression predicting PI, however, adding FPEO was associated with a substantial increase in explained variance (ΔR^2^ = 0.243), whereas the tested interaction effects were minimal. This pattern suggests that FPEO may warrant examination as a direct correlate or covariate in future prespecified structural models. Because FPEO includes behavior-proximal food-choice content, some conceptual proximity to PI and PEMCB remains possible despite acceptable HTMT values.

PS significantly moderated both hypothesized relationships, but the incremental contributions were modest (ΔR^2^ = 0.007 and 0.008). For the IB × PS interaction, the negative IB–PI association was statistically significant at mean and high levels of PS but not at low PS. One possible interpretation is that consumers who perceive environmental problems as more serious may scrutinize whether environmentally positioned food options are clearly defined, transparently labeled, and consistent with their expectations. This interpretation is tentative and should not be taken as evidence that perceived environmental severity causally increases resistance.

The AWFO × PS interaction was also statistically significant but small. Positive AWFO–SFDT associations were observed at low, mean, and high levels of PS and became somewhat stronger as PS increased. PS therefore appears to function as a limited contextual condition rather than as a dominant determinant of flexitarian responses.

Age-group differences were observed for six constructs, although the effect sizes were small to modest and practical scalar measurement invariance was supported. Respondents aged 46–61 years reported higher PI, PEMCB, AWFO, SFDT, and FPEO scores than both younger age groups, whereas PS did not differ significantly across age groups. These findings should be interpreted as age-group differences rather than generational or cohort effects because the comparisons were cross-sectional and exact birth year was not collected. Age, life stage, recruitment patterns, and other unmeasured characteristics may also have contributed to the observed differences. Accordingly, age group should be regarded as a supplementary source of consumer heterogeneity rather than as a primary explanation of flexitarian meal responses.

### 5.3. Theoretical and Practical Implications

The findings have three main theoretical implications. First, resistance-related and ethical/evaluative processes can be examined as complementary pathways without assuming that they form a single integrated causal mechanism. The IRT-informed pathway captures concerns related to processing, ambiguity, and labeling, whereas the VAB-informed ethical/evaluative pathway links animal-welfare-related food-choice orientation (AWFO) with support for flexitarian dietary transition (SFDT) and related consumer responses.

Second, the findings highlight the importance of behavioral-referent alignment in food-choice models. PI and PEMCB are related constructs but do not assess the same behavioral target, and SFDT represents support for dietary transition rather than a conventional personal attitude toward consuming flexitarian meals. Aligning construct labels with their actual item content therefore changes how the corresponding structural associations should be interpreted.

Third, FPEO and PS appear to play different roles. FPEO showed no significant moderation but exhibited a comparatively stronger direct association with PI in the hierarchical regression, whereas PS showed statistically detectable but practically small interaction effects. Future studies could test FPEO as a direct predictor or covariate within a prespecified latent-variable framework.

From a practical perspective, foodservice providers and marketers may consider testing whether clearer descriptions of ingredients, processing, labeling, and the meaning of flexitarian options reduce image-related concerns. Similarly, transparent and verifiable information about animal-welfare and ethical-production attributes may warrant testing among consumers for whom such considerations are salient. These are potential intervention directions rather than effects established by the present observational study.

Experimental and field evidence suggests that sustainability-related information can influence product perceptions and purchase intentions, but informational effects do not necessarily translate into actual reductions in meat consumption [63,64]. Future intervention studies should therefore distinguish changes in perceptions and intentions from changes in observed behavior.

Future research should also examine factors not measured here, including taste, price, availability, convenience, menu design, health motivations, social norms, and prior experience. Environmental orientations and age-group information may be useful for consumer segmentation, but neither should be considered in isolation. The modest interaction and age-group effect sizes suggest that AWFO, support for dietary transition, product-related concerns, and practical choice conditions may provide a more informative basis for future intervention testing.

### 5.4. Limitations and Future Research

Several limitations should be considered. First, the cross-sectional survey design permits estimation of contemporaneous associations among perceptions, food-choice orientations, supportive evaluations, intentions, and self-reported meal-choice tendencies, but it cannot establish temporal ordering or causal effects. Longitudinal studies, field experiments, behavioral choice tasks, and actual purchase records would provide stronger evidence regarding behavioral change.

Second, the use of non-probability convenience sampling through LINE, Facebook, student networks, and personal contacts limits population representativeness. Women accounted for 65.0% of the sample, and respondents aged 18–31 years accounted for 48.2%. Income was not collected, preventing assessment of socioeconomic representativeness on that dimension.

Third, all focal variables were measured by self-report in a single survey. Harman’s single-factor test and the poor fit of the single-factor CFA did not indicate dominance by a single common factor, but these diagnostics cannot exclude common-method or social-desirability bias. Such bias may be particularly relevant for environmentally normative constructs such as FPEO and PS.

Fourth, several constructs were short, adapted, or narrower than their conventional conceptual domains. IB showed marginal convergent validity (AVE = 0.479); AWFO represented a relatively narrow animal-welfare-related food-choice orientation rather than an abstract value construct; SFDT was not a conventional personal attitude scale; and PEMCB and FPEO contained behavior-proximal environmental content. Although the Fornell–Larcker criterion and HTMT ratios supported empirical discriminant validity, conceptual proximity among these measures should be considered when interpreting the findings. In addition, the latent SEM and composite-score moderation analyses differ in their treatment of measurement error; their coefficient magnitudes should therefore not be directly compared. Substantial multivariate non-normality was also detected, and the SEM was estimated using conventional maximum likelihood rather than a robust ML estimator. Although multiple fit indices and bias-corrected bootstrap confidence intervals were used to support interpretation, some residual sensitivity of the SEM estimates and standard errors to non-normality cannot be excluded.

Fifth, the questionnaire was administered in Chinese, and an English version was prepared for manuscript reporting and peer review. A formally documented back-translation procedure was not available. Several measures were context-adapted, and the study-specific description of flexitarian meals included examples that should not be interpreted as a universal taxonomy of flexitarian foods. Future studies should further validate translated and adapted measures and employ more standardized definitions of flexitarian meal choices across cultural settings.

Finally, respondents who reported consuming both vegetarian and non-vegetarian meals should not be classified as flexitarians solely on that basis because intentional meat reduction, meat-consumption frequency, and flexitarian self-identification were not assessed. Age-group comparisons, although supported by practical scalar measurement invariance, remain cross-sectional and may reflect age, life stage, recruitment patterns, or other unmeasured characteristics. The study was conducted in Taiwan, where animal-source food availability coexists with culturally established vegetarian practices; cross-cultural replication is needed before generalizing these resistance-related and ethical/evaluative associations to other food cultures.

## 6. Conclusions

This study examined consumer responses to flexitarian meals in Taiwan using complementary IRT-informed resistance and VAB-informed ethical/evaluative perspectives. Image-related concerns were modestly and negatively associated with purchase intention, whereas animal-welfare-related food-choice orientation (AWFO) was strongly associated with support for flexitarian dietary transition (SFDT). SFDT was positively associated with both purchase intention and self-reported pro-environmental meal-choice behavior (PEMCB), and bootstrap analyses indicated significant indirect associations of AWFO with both outcomes through SFDT. Although PI and PEMCB were positively correlated at the bivariate level, PI did not show a significant unique association with PEMCB after SFDT was included in the structural model. This distinction indicates that their zero-order covariation should not be interpreted as equivalent to a significant conditional structural association or attributed primarily to behavioral-referent differences.

FPEO did not significantly moderate the hypothesized relationships, although it showed a comparatively stronger direct association with purchase intention in the observed-composite regression analyses. PS significantly moderated the IB–PI and AWFO–SFDT associations, but the incremental variance explained by each interaction was less than 1%, indicating modest contextual effects. Practical scalar measurement invariance was supported across respondents aged 18–31, 32–45, and 46–61 years. The observed age-group differences were small to modest and should not be interpreted as generational or cohort effects.

Overall, the findings support the coexistence of resistance-related and ethical/evaluative associations in flexitarian meal choice and highlight the importance of aligning construct labels and interpretations with their actual measurement content. Given the cross-sectional convenience sample, adapted self-report measures, heterogeneous meal examples, and observed multivariate non-normality, the findings should be interpreted as associations rather than causal effects. Future experimental, longitudinal, and behavioral research should examine whether clearer product information, animal-welfare and ethical attributes, and practical choice conditions influence actual flexitarian meal selection and sustained meat reduction.

## Figures and Tables

**Table 1:** Characteristics of the study sample (*N* = 608).

Characteristic	Category	*n*	%
Sex	Male	213	35.0
	Female	395	65.0
Age (years)	18–21	31	5.1
	22–26	217	35.7
	27–31	45	7.4
	32–38	90	14.8
	39–45	60	9.9
	46–53	82	13.5
	54–61	83	13.7
Age group	18–31	293	48.2
	32–45	150	24.7
	46–61	165	27.1
Education	Junior high school or below	6	1.0
	Senior high/vocational school	67	11.0
	College or university	441	72.5
	Master’s degree or above	94	15.5
Dietary Preference	Vegetarian	28	4.6
	Non-vegetarian	275	45.2
	Consumes both vegetarian and non-vegetarian meals	305	50.2

Note. Age categories were recorded as an original variable in the dataset. The original age categories were combined into three broader groups (18–31, 32–45, and 46–61 years) for age-group comparisons. These groupings should not be interpreted as generational or cohort classifications because exact birth year was not collected. Dietary preference was not used to classify respondents as flexitarians. Income was not collected in the original questionnaire. Percentages may not sum to exactly 100% because of rounding.

**Table 2:** Measurement constructs, items, and sources.

Construct/Variable	Number of Statements	Measuring Items	Sources
Image-Related Concerns (IB)	3	IB1. I believe that flexitarian meals may contain more processed ingredients. IB2. I think the guidelines for flexitarian meals are vague and hard to understand. IB3. I have concerns regarding the ingredient labeling of flexitarian meals.	[48]
Purchase Intention (PI)	5	PI1. If given the option, I would like to choose to consume flexitarian meals. PI2. If given the option, I would be interested in purchasing flexitarian meals. PI3. If given the option, I would like to choose to consume flexitarian meals in the next two weeks. PI4. If given the option, I think I would be very willing to purchase flexitarian meals. PI5. In future meal choices, I would like to prioritize purchasing flexitarian meals as much as possible.	[49,50]
Pro-Environmental Meal-Choice Behavior (PEMCB)	2	PB1. When purchasing flexitarian meals, I always choose options that have a lower environmental impact. PB2. When choosing among similar meals, I usually opt for those with a lower environmental impact.	[51]
Animal-Welfare-Related Food-Choice Orientation (AWFO)	3	AWV 1. I care about animal welfare, so I purchase brands that are labeled as not involving animal slaughter. AWV 2. I care about animal welfare, so I choose brands and meals that do not involve animal testing. AWV 3. I care about animal welfare, so I prefer to buy meals that are ethically produced.	[52]
Support for Flexitarian Dietary Transition (SFDT)	2	AT1. I believe that more promotional activities for flexitarian diets are needed in Taiwan. AT2. I believe it is important to address issues caused by meat consumption in Taiwan.	[53]
Food-Related Pro-Environmental Orientation (FPEO)	3	EC1. Due to environmental concerns, I try to reduce my purchases of meat-based meals that harm the environment. EC2. Due to environmental concerns, I actively seek out environmentally friendly meal options. EC3. Due to environmental concerns, I try to encourage my family and friends to reduce their purchases of meals that harm the environment.	[54]
Perceived Environmental Severity (PS)	4	PS1. I believe that environmental damage has significant negative impacts on the economy and society. PS2. I believe that environmental damage is dangerous to the environment and can lead to issues such as global warming. PS3. I believe that the severity of environmental damage is increasing. PS4. I believe that environmental damage is occurring with greater frequency.	[55]

**Table 3:** Factor loadings, reliability, and convergent validity.

Items	Factor Loading	Cronbach’s α	CR	AVE
Image-Related Concerns (IB)		0.729	0.732	0.479
IB 1.	0.715			
IB 2	0.603			
IB 3	0.749			
Purchase Intention (PI)		0.925	0.925	0.711
PI 1	0.855			
PI 2	0.842			
PI 3	0.806			
PI 4	0.867			
PI 5	0.846			
Pro-Environmental Meal-Choice Behavior (PEMCB)		0.882	0.882	0.789
PB 1	0.888			
PB 2	0.888			
Animal-Welfare-Related Food-Choice Orientation (AWFO)		0.878	0.879	0.708
AWV 1	0.855			
AWV 2	0.846			
AWV 3	0.823			
Support for Flexitarian Dietary Transition (SFDT)		0.766	0.770	0.627
AT 1	0.835			
AT 2	0.746			
Food-Related Pro-Environmental Orientation (FPEO)		0.857	0.858	0.669
EC 1	0.782			
EC 2	0.846			
EC 3	0.825			
Perceived Environmental Severity (PS)		0.928	0.926	0.759
PS 1	0.790			
PS 2	0.819			
PS 3	0.937			
PS 4	0.928			

Note: IB = Image-Related Concerns; PI = Purchase Intention; PEMCB = Pro-Environmental Meal-Choice Behavior; AWFO = Animal-Welfare-Related Food-Choice Orientation; SFDT = Support for Flexitarian Dietary Transition; FPEO = Food-Related Pro-Environmental Orientation; PS = Perceived Environmental Severity; CR = Composite Reliability; AVE = Average Variance Extracted. The original item codes PB, AWV, AT, and EC were retained for traceability.

**Table 4:** Means, Standard Deviations, and Pearson Correlations Based on Observed Composite Scores.

Construct	Mean	S.D.	1	2	3	4	5	6	7
1. IB	4.554	1.225	1.00						
2. PI	4.005	1.366	−0.130 **	1.00					
3. PEMCB	4.451	1.494	−0.004	0.526 **	1.00				
4. AWFO	4.497	1.430	−0.053	0.566 **	0.602 **	1.00			
5. SFDT	4.577	1.361	−0.034	0.626 **	0.524 **	0.607 **	1.00		
6. FPEO	4.164	1.505	0.088 *	0.480 **	0.525 **	0.493 **	0.332 **	1.00	
7. PS	5.514	1.294	0.113 **	0.341 **	0.467 **	0.472 **	0.497 **	0.162 **	1.00

Note: *N* = 608. Values are Pearson correlations based on observed composite scores. * *p* < 0.05; ** *p* < 0.01.

**Table 5:** Structural Path Estimates and Bootstrap Indirect Associations.

**Panel A. Direct Structural Paths**							
**Hypothesis**	**Path**	**B**	**SE**	**β**	**Bias-Corrected 95% CI for β**	***p* (ML)**	**Result**
H1	IB → PI	−0.152	0.043	−0.128	[−0.202, −0.053]	<0.001	Supported
H2	AWFO → SFDT	0.676	0.043	0.808	[0.748, 0.859]	<0.001	Supported
H3	PI → PEMCB	0.041	0.076	0.041	[−0.141, 0.224]	0.588	Not supported
H4	SFDT → PEMCB	0.833	0.107	0.695	[0.508, 0.877]	<0.001	Supported
H5	SFDT → PI	0.925	0.061	0.780	[0.724, 0.832]	<0.001	Supported
**Panel B. Standardized Indirect Associations**							
**Indirect Association**		**Standardized Indirect Effect**			**Bias-Corrected 95% CI**		
AWFO → SFDT → PI		0.630			[0.563, 0.695]		
AWFO → SFDT → PEMCB		0.562			[0.400, 0.719]		
SFDT → PI → PEMCB		0.032			[−0.112, 0.172]		
AWFO → SFDT → PI → PEMCB		0.026			[−0.092, 0.138]		
AWFO → PEMCB (total indirect)		0.588			[0.499, 0.669]		

Note. B = unstandardized coefficient; SE = standard error; β = standardized coefficient. The reported *p*-values are based on maximum-likelihood parameter estimates, whereas the bias-corrected 95% confidence intervals are based on 5000 bootstrap resamples. Indirect associations are interpreted statistically and do not imply causal mediation because of the cross-sectional design.

**Table 6:** Moderating role of food-related pro-environmental orientation in the association between image-related concerns and purchase intention.

Variables	PI		
	Model 1	Model 2	Model 3
**Step 1: Independent variable** IB	−0.130 **	−0.173 ***	−0.173 ***
**Step 2: Moderator** FPEO		0.495 ***	0.493 ***
**Step 3: Interaction** IB × FPEO			−0.037
R^2^	0.017	0.260	0.261
ΔR^2^	0.017	0.243	0.001
ΔF	10.341 **	198.806 ***	1.109

Note. Entries are standardized regression coefficients (β) from observed-composite OLS models. The outcome, focal predictor, and moderator composite scores were standardized as z scores before estimation, and interaction terms were formed as products of the corresponding standardized predictor and moderator. The overall model degrees of freedom were F(1, 606), F(2, 605), and F(3, 604) for Models 1–3, respectively. Each ΔF test involved one additional predictor. ** *p* < 0.01; *** *p* < 0.001.

**Table 7:** Moderating role of food-related pro-environmental orientation in the association between animal-welfare-related food-choice orientation and support for flexitarian dietary transition.

Variables	SFDT		
	Model 1	Model 2	Model 3
**Step 1: Independent variable** AWFO	0.607 ***	0.586 ***	0.588 ***
**Step 2: Moderator** FPEO		0.043	0.048
**Step 3: Interaction** AWFO × FPEO			0.044
R^2^	0.369	0.370	0.372
ΔR^2^	0.369	0.001	0.002
ΔF	354.093 ***	1.333	1.821

Note. Entries are standardized regression coefficients (β) from observed-composite OLS models. The outcome, focal predictor, and moderator composite scores were standardized as z scores before estimation, and interaction terms were formed as products of the corresponding standardized predictor and moderator. The overall model degrees of freedom were F(1, 606), F(2, 605), and F(3, 604) for Models 1–3, respectively. Each ΔF test involved one additional predictor. *** *p* < 0.001.

**Table 8:** Moderating role of perceived environmental severity in the association between image-related concerns and purchase intention.

Variables	PI		
	Model 1	Model 2	Model 3
**Step 1: Independent variable** IB	−0.130 **	−0.170 ***	−0.164 ***
**Step 2: Moderator** PS		0.360 ***	0.345 ***
**Step 3: Interaction** IB × PS			−0.087 *
R^2^	0.017	0.145	0.152
ΔR^2^	0.017	0.128	0.007
ΔF	10.341 **	90.548 ***	5.199 *

Note. Entries are standardized regression coefficients (β) from observed-composite OLS models. The outcome, focal predictor, and moderator composite scores were standardized as z scores before estimation, and interaction terms were formed as products of the corresponding standardized predictor and moderator. The overall model degrees of freedom were F(1, 606), F(2, 605), and F(3, 604) for Models 1–3, respectively. Each ΔF test involved one additional predictor. * *p* < 0.05; ** *p* < 0.01; *** *p* < 0.001.

**Table 9:** Moderating role of perceived environmental severity in the association between animal-welfare-related food-choice orientation and support for flexitarian dietary transition.

Variables	SFDT		
	Model 1	Model 2	Model 3
**Step 1: Independent variable** AWFO	0.607 ***	0.480 ***	0.458 ***
**Step 2: Moderator** PS		0.270 ***	0.316 ***
**Step 3: Interaction** AWFO × PS			0.096 **
R^2^	0.369	0.426	0.433
ΔR^2^	0.369	0.057	0.008
ΔF	354.093 ***	59.775 ***	8.099 **

Note. Entries are standardized regression coefficients (β) from observed-composite OLS models. The outcome, focal predictor, and moderator composite scores were standardized as z scores before estimation, and interaction terms were formed as products of the corresponding standardized predictor and moderator. The overall model degrees of freedom were F(1, 606), F(2, 605), and F(3, 604) for Models 1–3, respectively. Each ΔF test involved one additional predictor. ** *p* < 0.01; *** *p* < 0.001.

**Table 10:** Age-Group Differences in Observed Composite Scores and Scheffé Post Hoc Comparisons.

Construct	18–31 Years (*n* = 293), M (SD)	32–45 Years (*n* = 150), M (SD)	46–61 Years (*n* = 165), M (SD)	F(2, 605)	*p*	η^2^	Significant Scheffé Comparisons
Image-Related Concerns (IB)	4.315 (1.107)	4.853 (1.162)	4.707 (1.393)	11.732	<0.001	0.037	32–45 > 18–31; 46–61 > 18–31
Purchase Intention (PI)	3.902 (1.195)	3.820 (1.536)	4.355 (1.429)	7.811	<0.001	0.025	46–61 > 18–31; 46–61 > 32–45
Pro-Environmental Meal-Choice Behavior (PEMCB)	4.157 (1.349)	4.380 (1.571)	5.036 (1.510)	19.634	<0.001	0.061	46–61 > 18–31; 46–61 > 32–45
Animal-Welfare-Related Food-Choice Orientation (AWFO)	4.422 (1.254)	4.318 (1.546)	4.792 (1.573)	5.156	0.006	0.017	46–61 > 18–31; 46–61 > 32–45
Support for Flexitarian Dietary Transition (SFDT)	4.524 (1.262)	4.307 (1.418)	4.918 (1.416)	8.575	<0.001	0.028	46–61 > 18–31; 46–61 > 32–45
Food-Related Pro-Environmental Orientation (FPEO)	4.049 (1.495)	3.867 (1.468)	4.638 (1.455)	12.434	<0.001	0.039	46–61 > 18–31; 46–61 > 32–45
Perceived Environmental Severity (PS)	5.422 (1.267)	5.530 (1.208)	5.664 (1.404)	1.870	0.155	0.006	n.s.

Note. Values are means (M) and standard deviations (SD) of observed composite scores calculated as the arithmetic mean of the retained items within each construct. η^2^ = eta-squared. Scheffé comparisons are reported only for statistically significant pairwise differences. n.s. = not significant. The three groups represent age categories rather than generational or birth-cohort classifications.

## Data Availability

The data presented in this study are available from the corresponding author upon reasonable request.

## Supplementary Materials

The following supporting information can be downloaded at: https://www.mdpi.com/article/10.3390/foods15203675/s1, Table S1: Heterotrait-Monotrait Ratios (HTMT) Among Study Constructs; Table S2: Measurement Invariance Across the Three Age Groups; Table S3: Detailed CFA Measurement-Model Estimates and Latent Construct Correlations; Table S4: Item-Level Descriptive Statistics for the Final 22 Measurement Items.
