# Supplementary material for: Consumer Responses to Flexitarian Meals in Taiwan: Resistance-Based and Ethical–Evaluative Pathways
Source: Foods. 2026 Oct 16;15(20):3675. doi: 10.3390/foods15203675 (PMC13703642; doi:10.3390/foods15203675)
Supplement: Supplementary file 1 [file foods-15-03675-s001.zip › foods-4593909-supplementary.pdf]

Supplementary Materials

Table S1. Heterotrait-Monotrait Ratios (HTMT) Among Study Constructs.

Construct	IB	PI	PEMCB	AWFO	SFDT	FPEO	PS
IB							
PI	0.157						
PEMCB	0.029	0.583					
AWFO	0.068	0.628	0.684				
SFDT	0.044	0.745	0.637	0.740			
FPEO	0.112	0.539	0.604	0.568	0.411		
PS	0.137	0.368	0.515	0.523	0.590	0.181	

Note. Values are shown in the lower triangle. All HTMT ratios were below 0.85; the largest was PI–SFDT = 0.745. IB = Image-Related Concerns; PI = Purchase Intention; PEMCB = Pro-Environmental Meal-Choice Behavior; AWFO = Animal-Welfare-Related Food-Choice Orientation; SFDT = Support for Flexitarian Dietary Transition; FPEO = Food-Related Pro-Environmental Orientation; PS = Perceived Environmental Severity.

Table S2. Measurement Invariance Across the Three Age Groups

Model	χ^2	df	CFI	TLI	RMSEA	ΔCFI	ΔRMSEA	$\Delta\chi^2$ (Δdf)	p
Configural	1341.544	564	0.921	0.902	0.048	—	—	—	—
Metric	1407.271	594	0.917	0.903	0.047	0.004	0.001	65.727 (30)	<0.001
Scalar	1458.383	624	0.915	0.905	0.047	0.002	<0.001	51.112 (30)	0.009

Note. ΔCFI and ΔRMSEA are reported as absolute changes from the less constrained model. Although chi-square difference tests were significant for the metric and scalar constraints, the small changes in CFI and RMSEA supported practical measurement invariance. The three groups were constructed from the original age-category variable: 18–31 years (n = 293), 32–45 years (n = 150), and 46–61 years (n = 165). Because exact birth year was not collected, these groupings should be interpreted as age groups rather than generational or birth-cohort classifications.

Table S3. Detailed CFA Measurement-Model Estimates and Latent Construct Correlations.

Panel A. Item-level CFA estimates.

Construct	Item	Standardized loading	Unstandardized B	SE	C.R.	p
IB	IB1	.715	1.000 (fixed)	—	—	—
IB	IB2	.603	.815	.071	11.488	<0.001
IB	IB3	.749	.991	.083	12.012	<0.001
PI	PI1	.855	1.000 (fixed)	—	—	—
PI	PI2	.842	.993	.038	26.478	<0.001
PI	PI3	.806	.962	.039	24.619	<0.001
PI	PI4	.867	1.003	.036	27.844	<0.001
PI	PI5	.846	.946	.035	26.687	<0.001
PEMCB	PB1	.888	1.000 (fixed)	—	—	—
PEMCB	PB2	.888	1.050	.041	25.416	<0.001
AWFO	AWV1	.855	1.000 (fixed)	—	—	—
AWFO	AWV2	.846	.972	.039	24.948	<0.001
AWFO	AWV3	.823	1.003	.042	24.005	<0.001
SFDT	AT1	.835	1.000 (fixed)	—	—	—
SFDT	AT2	.746	.951	.051	18.515	<0.001
FPEO	EC1	.782	1.000 (fixed)	—	—	—
FPEO	EC2	.846	1.088	.052	21.002	<0.001
FPEO	EC3	.825	1.062	.052	20.576	<0.001
PS	PS1	.790	1.000 (fixed)	—	—	—
PS	PS2	.819	1.013	.045	22.745	<0.001
PS	PS3	.937	1.159	.042	27.284	<0.001
PS	PS4	.928	1.138	.042	26.942	<0.001

Note. The first indicator of each latent construct was fixed to 1.000 for scale identification. Original questionnaire item codes PB, AWV, AT, and EC are retained for traceability, while the corresponding construct labels reported in the manuscript are PEMCB, AWFO, SFDT, and FPEO.

Panel B. CFA latent construct correlations.

Construct	IB	PI	PEMCB	AWFO	SFDT	FPEO	PS
IB							
PI	-0.159						
PEMCB	0.000	0.587					
AWFO	-0.066	0.631	0.683				
SFDT	-0.034	0.751	0.631	0.731			
FPEO	0.120	0.530	0.602	0.554	0.407		
PS	0.135	0.356	0.483	0.491	0.569	0.182	

Note. Values are shown in the lower triangle. The largest latent correlation was PI–SFDT = 0.751. The corresponding observed composite-score Pearson correlation was 0.626; these are distinct quantities from different levels of analysis.

Table S4. Item-Level Descriptive Statistics for the Final 22 Measurement Items.

Construct	Item	N	Mean	SD
IB	IB1	608	4.607	1.567
IB	IB2	608	4.605	1.513
IB	IB3	608	4.451	1.483
PI	PI1	608	4.166	1.565
PI	PI2	608	4.222	1.579
PI	PI3	608	3.773	1.596
PI	PI4	608	4.115	1.549
PI	PI5	608	3.747	1.496
PEMCB	PB1	608	4.480	1.541
PEMCB	PB2	608	4.421	1.619
AWFO	AWV1	608	4.498	1.582
AWFO	AWV2	608	4.707	1.553
AWFO	AWV3	608	4.285	1.650
SFDT	AT1	608	4.760	1.464
SFDT	AT2	608	4.395	1.558
FPEO	EC1	608	4.230	1.700
FPEO	EC2	608	4.192	1.709
FPEO	EC3	608	4.069	1.711
PS	PS1	608	5.342	1.455
PS	PS2	608	5.508	1.421
PS	PS3	608	5.597	1.420
PS	PS4	608	5.609	1.409

Note. All items were rated on seven-point Likert scales ranging from 1 (strongly disagree) to 7 (strongly agree). N = 608 for every item. Original questionnaire item codes PB, AWV, AT, and EC are retained for correspondence with the source dataset, while the corresponding construct labels reported in the manuscript are PEMCB, AWFO, SFDT, and FPEO.
